# Trace Metals in Pork Meat Products Marketed in Italy: Occurrence and Health Risk Characterization

**DOI:** 10.1007/s12011-020-02417-z

**Published:** 2020-11-12

**Authors:** Grazia Barone, Arianna Storelli, Nicoletta C. Quaglia, Rita Garofalo, Daniela Meleleo, Antonio Busco, Maria Maddalena Storelli

**Affiliations:** 1 Biosciences, Biotechnology and Biopharmaceutical Department, University of Bari, Strada Prov.le per Casamassima Km 3, 70010 Valenzano (BA), Italy; 2 Department of Emergency and Organ Transplant, University of Bari “Aldo Moro”, Strada Prov.le per Casamassima Km 3, 70010 Valenzano (BA), Italy

**Keywords:** Carcinogenic and noncarcinogenic risk, Hazard index, Margin of exposure, Pork meat products, Trace metals

## Abstract

This study provides valuable information on the levels of various trace metals (Pb, Cd, Hg, Zn, Cu, Cr) in meat products (baked ham, raw ham, mortadella, cured sausage, würstel, salami) from South Italy and calculates potential health risk toxicity associated with their consumption for the total population and for children. In the samples studied metal concentrations are within the permissible legal limits (Cd: 0.01–0.03 μg g^−1^ w.w., Hg: 0.01–0.02 μg g^−1^ w.w., Zn: 5.71–7.32 μg g^−1^ w.w., Cu: 1.08–1.21 μg g^−1^ w.w., Cr: 0.15–0.23 μg g^−1^ w.w.), except for Pb (Pb: 0.22–0.38 μg g^−1^ w.w.). The estimated intake values are within the provisional tolerable daily intake limits for toxic metals and recommended daily intake values for essential metals in both tested groups. The noncarcinogenic risk values of the individual metals indicate that there is no health risk, but their combined effects might constitute a potential risk for children. Furthermore, the cumulative cancer risk of all samples studied exceeds the recommended threshold risk limit (> 10^−4^) in both total population and children, indicating a risk of potential health problems for consumers especially for children, who are more vulnerable to toxic metal exposure.

## Introduction

Over the past few decades, changes in life and food style of population have led an increase in the demand for processed foods [1]. Meat is a major segment of processed food, with pork meat having the greatest economic dimension. Pork meat products are, in fact, the most consumed in the world, either in the fresh or processed form [2]. In Italy, the most commonly consumed processed meats are baked ham in the first place, with a percentage equal to 26.3%, followed by raw ham with 22.0%, mortadella and würstel up to 19.0%, salami with 7.8%, and other cured meats making 23.7% [2]. Today, there is a considerable concern over the extent of environmental pollution with chemical contaminants and their relationship to public health. Among these substances, pollution by heavy metals is recognized as one of the most dangerous problems in the world, and of primary concern for proven hazardous nature are mercury, lead, and cadmium. Consumption of food contaminated by these elements can, in fact, cause profound biochemical and neurological changes in the human body determining serious health risks. In particular, neurological and behavioral disorders may be observed after exposure to mercury, with symptoms including primarily tremors, insomnia, memory loss, neuromuscular effects, headaches, and cognitive and motor dysfunction [3]. A number of diseases, especially neurological are also associated with the excess of Pb concentration in food, in addition to its implication in causing cardiovascular problems [4], while exposure to Cd determines numerous adverse effects, including impaired kidney function, poor reproductive capacity, fragile bones, hypertension, and tumors [5–8]. However, not only toxic elements but also essential elements due to their role within the human metabolism present an intriguing relation with health, showing either nutritional or toxicological effects [9, 10]. At high concentrations, Cr causes nephritis, anuria, and extensive lesions in kidneys [8] and critical doses of Cu can cause inflammation in brain tissue, anorexia, hair loss, depression, kidney and liver dysfunction, learning disorders, autism, and cancer [7]. Also zinc, responsible for some important biological functions at high levels, above the limit of 50 μg g^−1^, is harmful to human health causing a negative impact on the gastrointestinal system [11]. Nevertheless, only three metals, lead, cadmium, and mercury have been included in the European Union regulation establishing maximum permitted levels (MPLs) in a wide range of foodstuffs [12–14]. For mercury, in particular, the legislation stipulates maximum levels solely for the muscle fish meat, with the assumption that the remaining food groups do not pose a threat to human health. However, in the contest of food safety the levels, not only for toxic metals but also for other trace elements need to be accurately controlled The available literature has, principally, approached the determination of toxic metals in meat [15–22], while fewer are those dedicated to processed meat [23–28]. This is particularly true for Italy where the data collection on this topic was done in the Northern part of the Italian peninsula [29–31], while to the best of our knowledge, the Southern one have never been considered before. As a consequence of what have been mentioned above, this study was intended to augment the currently available information, through the following objectives: (1) to analyze the content of trace elements (mercury, cadmium, lead, chromium, copper, and zinc) in processed pork meat products sampled in Italy; (2) to check that the measured concentrations are below the maximum permitted levels by the current European legislation; and (3) to estimate the exposure and health risk either for total population or for children, segment of population extensively vulnerable to the toxicant exposure.

## Materials and Methods

### Sample Collection

A total number of 100 samples of processed pork meats, including baked ham (no. 15 samples), raw ham (no. 16 samples), mortadella (no. 15 samples), cured sausage (no. 16 samples), würstel (no. 18 samples), and salami (no. 20 samples) were randomly collected from September till December 2018 in most important commercial centers of Apulian region in South Italy. All collected samples were placed in clear polythene bags according to their type and brought to the laboratory for preparation and analysis. About 200 g from each product were homogenized or finely chopped and stored at − 20 °C. After thawed, accurately weighted aliquots of about 2 g of each product was taken and examined for the presence of residues of metals. Prior to use, all the plastic material used for storing and treating was soaked in diluted nitric acid for 24 h and then rinsed with distilled deionized water.

### Chemical Analyses

The extractive analytical procedure and the instrumental conditions for determine metal concentrations have been described in detail elsewhere [32]. Briefly, aliquots of the samples were digested to a transparent solution with a mixture of HNO_3_-HClO_4_ (8:3) for Cd, Pb, Zn, Cu, and Cr determination and with a mixture of H_2_SO_4_–HNO_3_ (1:1) for Hg. The completely digested samples were allowed to cool and diluted with deionized water according to the method recommended by the Official Italian Agencies [33]. The metals content was determined by an atomic absorption spectrophotometer (AA 7000 Shimadzu, Milan, Italy). Zn was analyzed by flame, Cd, Pb, Cr, and Cu by using a graphite furnace (pyrocoated tube) (GFA-7000) and Hg by using a hydride vapor generator (HVG-1) after reduction by NaBH_4_.

### Quality Control and Assurance

Certified reference material (SRM1577C Bovine Liver NIST National Institute of Standards and Technology, USA) was treated and analyzed in the same way as the samples. Results (Hg: 5.15 ± 0.01 μg kg^−1^; Cd: 93.0 ± 0.05 μg kg^−1^; Pb: 60.3 ± 1.3 μg kg^−1^; Cr: 49.0 ± 10.0 μg kg^−1^; Zn: 176.0 ± 11.0 mg kg^−1^; Cu: 293.0 ± 6.3 mg kg^−1^ dry weight) were in good agreement with the certified values (Hg: 5.36 ± 0.17 μg kg^−1^; Cd: 97.0 ± 1.4 μg kg^−1^; Pb: 62.8 ± 1.0 μg kg^−1^; Cr: 53.0 ± 14.0 μg kg^−1^; Zn: 181.1 ± 1.0 mg kg^−1^; Cu: 275.2 ± 4.6 mg kg^−1^ dry weight) and the standard deviation was low (*n* = 3), proving the good repeatability of the methods. The results for the standard reference material displayed recoveries of the elements ranging from 93 to 108% (*n* = 3). The limit of detection (LOD) (Hg: 5; Cd: 0.12; Pb: 10; Zn: 24; Cu: 26; Cr: 5 ng g^−1^ wet weight) is defined as the concentration corresponding to three times the standard deviation of blanks and the standards of quantification (LOQ) are the following: Hg: 13; Cd: 0.40; Pb: 0.38; Zn: 87; Cu: 81; Cr: 16 ng g^−1^ wet weight. Two blank samples were analyzed together with each sample batch. Metal concentrations in blanks were below the detection limits in all the analyses. Blanks and calibration standard solutions were analyzed in a similar way to the digested sample solution, and calibration curves were constructed. Analyses were duplicated to check the reproducibility of the results. Relative standard deviations among replicates were always less than 10%. Recovery tests were performed for the investigated metals in selected samples by spiking analyzed samples with aliquots of the metal standards and then carrying out digestion. The recovery percentages ranged from 96 to 99%.

### Risk Assessment

Estimated daily intake (EDI) of Hg, Cd, Pb, Cr, Cu, and Zn through pork meat product consumption was calculated using the following equation:

$$ \mathrm{EDI}=\left(C\times \mathrm{IR}\right)/\mathrm{BW} $$

where.

*C*: is the element concentration in samples studied,
IR: is the daily ingestion rate (g day^−1^) (meat product consumption total population: 27.0 g day^−1^) [34], (meat product consumption children: 23.3 g day^−1^) [35],
WAB: is the adult average body weight (70 kg).
WAB: is the children average body weight (26.1 kg).

The EDIs were compared with the European Food Safety Authority limits expresses as provisional tolerable weekly intake (PTWI) for Hg and Pb and as provisional tolerable monthly intake (PTMI) for Cd [36–38], which represent an estimation of the amount of substance that can be ingested over a lifetime without appreciative risk. In case of essential metals, the estimated ingestions were compared with the average requirement values of RDA [39]. In addition, the EDI values obtained have been used to estimate the noncancer and cancer risk. Noncancer risk was estimated through the following indicators:

Hazard quotient (HQ) determines the potential risk caused by metals individually and is described by the following equation:

$$ \mathrm{HQ}=\mathrm{EFr}\times \mathrm{ED}\times \mathrm{FIR}\times \mathrm{C}/\mathrm{RfD}\times \mathrm{WAB}\times \mathrm{AT}\times {10}^{-3} $$

where.

EFr: is the exposure frequency (365 days/year),
ED: is the exposure duration (70 years), equivalent to the average lifetime,
FIR: is the food ingestion rate (total population: 27.0 g day^−1^; children: 23.3 g day^−1^),
C: is the metal concentration,
RfD: is the oral reference dose (oral reference dose: Pb = 3.6 × 10^−2^ mg kg^−1^ day^−1^, Cd = 1.0 × 10^−3^ mg kg^−1^ day^−1^, Hg = 1.0 × 10^−4^ mg kg^−1^ day^−1^, Cr(VI) = 3.0 × 10^−3^ mg kg^−1^ day^−1^, Cu = 4.0 × 10^−2^ mg kg^−1^ day^−1^, Zn = 3.0 × 10^−1^ mg kg^−1^ day^−1^ [40, 41],
WAB: is the adult average body weight (70 kg),
WAB: is the children average body weight (26.1 kg),
AT: is the averaging exposure time for non-carcinogens (365 days/year × ED).

Hazard index (HI) determines the potential risk triggered by metals collectively and is calculated as sum of HQ.

$$ \mathrm{HI}={\mathrm{HQ}}_{\mathrm{Pb}}+{\mathrm{HQ}}_{\mathrm{Cd}}+\kern1.5em {\mathrm{HQ}}_{\mathrm{n}.\dots } $$

If the HI value obtained is under “1,” an adverse effect is out of the question in terms of human health.

The possibility of cancer risks in the studied meat products through intake of carcinogenic heavy metals (Pb, Cd, and Cr) was estimated using the incremental lifetime cancer risk (ILCR) [42] and calculated using the following equation:

$$ \mathrm{ILCR}=\mathrm{CDI}\ \mathrm{x}\ \mathrm{CSF} $$

where.

CSF: is the cancer slope factor of the metals considered [slope factor: Pb = 0.0085 mg kg^−1^ day^−1^, Cd = 15 mg kg^−1^ day^−1^ [31], Cr = 0.5 mg kg^−1^ day^−1^ [41],

CDI (chronic daily intake of chemical, mg kg^−1^ bw day^−1^) represents the lifetime average daily dose of exposure to the chemical, calculated on the basis of following equation:

$$ \mathrm{CDI}=\left(\mathrm{EDI}\times \mathrm{EFr}\times \mathrm{EDtot}\right)/\mathrm{AT} $$

where

EDI: is the estimated daily intake,
EFr: is the exposure frequency (365 days/year),
EDtot: is the exposure duration (70 years), equivalent to the average lifetime,
AT: is the averaging exposure time for noncarcinogens (365 days/year × ED).

The cumulative cancer risk as a result of exposure to multiple cancer heavy metals was assumed to be the sum of the individual heavy metal increment risks.

The Pb risk of dietary exposure was also calculated according to the margin of exposure (MOE) approach [30] as recommended by the European Food safety Authority (EFSA) to assess the risk caused by substances that are both genotoxic and carcinogenic [43, 44] using the following equation:.

$$ \mathrm{MOE}=\mathrm{BMDL}/\mathrm{EDI} $$

where BMDL is the benchmark dose (lower confidence limit) estimated at 1.2 and 0.6 μg kg^−1^ b. w. day^−1^ for adult and children, respectively [45].

A MOE of less than 1 indicates a high health risk whereas a MOE greater than 1 indicates an acceptable low risk [46, 47].

### Statistical Analysis

Kruskal–Wallis and Mann–Whitney tests were used to test the hypothesis about concentration differences as a function of processed meat types and to determine whether there were differences in the levels of metal accumulation. All analyses were determined at significance levels of *P* < 0.05.

## Results and Discussion

All obtained results, subdivided by product category and presented as minimum, maximum and average values ± standard deviation are illustrated in Table 1. In addition in the same table, the maximum permitted limits for trace metals tested have been reported. There is a paucity of earlier literature concerning the processed meats to compare the present findings and in addition many studies report the concentrations on dry weight basis. However, an overview of levels expressed in fresh weight basis in processed pork meats worldwide is shown in Table 2.

**Table 1:** Metal concentrations (μg g^−1^ wet weight) (range, mean ± standard deviation) and maximum permitted limits in pork meat products

Meat products	Pb	Cd	Hg	Zn	Cu	Cr
Baked ham	0.18–0.31	ND–0.03	ND-0.03	5.75–7.55	0.85–1.32	0.07–0.22
	0.22 ± 0.04	0.01 ± 0.01	0.01 ± 0.01	6.02 ± 0.46	1.08 ± 0.15	0.15 ± 0.05
Raw ham	0.16–0.55	ND–0.04	ND–0.05	4.22–7.36	0.96–1.33	0.13–0.25
	0.30 ± 0.12	0.01 ± 0.01	0.01 ± 0.02	5.71 ± 1.08	1.11 ± 0.10	0.19 ± 0.04
Mortadella	0.21–0.58	ND–0.05	ND–0.05	4.56–8.16	0.89–1.35	0.15–0.27
	0.34 ± 0.10	0.02 ± 0.02	0.02 ± 0.02	6.03 ± 0.96	1.13 ± 0.14	0.20 ± 0.04
Cured sausage	0.17–0.59	ND–0.05	ND–0.05	4.58–9.05	0.87–1.25	0.14–0.31
	0.34 ± 0.14	0.03 ± 0.02	0.01 ± 0.02	6.16 ± 1.69	1.11 ± 0.12	0.23 ± 0.05
Würstel	0.24–0.59	ND–0.05	ND–0.05	4.56–9.23	1.05–1.31	0.13–0.32
	0.38 ± 0.11	0.03 ± 0.02	0.02 ± 0.02	6.05 ± 1.14	1.21 ± 0.08	0.22 ± 0.07
Salami	0.16–0.49	ND–0.05	ND–0.05	5.01–10.18	0.85–1.35	0.11–0.29
	0.29 ± 0.11	0.02 ± 0.02	0.01 ± 0.02	7.32 ± 1.70	1.13 ± 0.16	0.20 ± 0.05
Maximum permitted limits	0.10^a^	0.05^b^	0.05^c^	50^d^	3^d^	1.0^e^

^a^[12]; ^b^[13]; ^c^[48–50]; ^d^[51]; ^e^[49]

**Table 2:** Results obtained for trace elements in pork meat products from Italy compared with reported literature values

Meat products	Origin	Hg	Cd	Pb	Cr	Cu	Zn	References
Baked ham	Italy	ND–0.03 0.01	ND–0.05 0.01	0.18–0.28 0.22	0.07–0.22 0.15	0.85–1.32 1.08	5.75–7.55 6.02	This study
	Spain	–	BDL	0.01–0.02 0.02	0.08–0.13 0.10	0.53–0.76 0.60	–	Marin et al. [52]
	Spain	0.011	0.000	0.026	–	–	–	Martorell et al. [53]
	Spain^a^	< 0.100	< 0.025	0.044	–	–	–	Martí-Cid et al. [54]
	Romania	–	0.09–0.16 0.13	0.35–0.86 0.65	–	0.65–0.92 0.73	24.6–62.1 33.5	Hoha et al. [1]
	Slovak Republic	0.005–0.007 0.006	–	–	–	–	–	Lukáčová et al. [55]
	Iran	–	–	0.20–0.50 0.32	–	1.20–2.00 1.48	1.13–5.77 3.18	Sobhanardakani [56]
Raw ham	Italy	ND–0.05 0.01	ND–0.04 0.01	0.16–0.55 0.30	0.13–0.25 0.19	0.96–1.33 1.11	4.22–7.36 5.71	This study
	Spain	0.009	0.006	0.172	–	–	–	Martorell et al. [53]
Mortadella	Italy	ND–0.05 0.02	ND–0.05 0.02	0.25–0.52 0.34	0.15–0.27 0.20	0.89–1.35 1.13	4.56–8.16 6.03	This study
Cured sausage	Italy	ND–0.05 0.01	ND–0.05 0.03	0.17–0.59 0.34	0.15–0.31 0.23	0.87–1.25 1.11	4.58–9.05 6.16	This study
	Spain	–	0.01–0.01 0.01	0.04–0.07 0.06	0.08–0.36 0.13	1.00–1.31 1.13	–	Marin et al. [52]
	Spain	0.005	0.006	0.08	–	–	–	Martorell et al. [53]
	Turkey	–	BDL	0.10	BDL	0.18	13.0	Daşbaşi et al. [57]
	Turkey	–	0.008	0.13	0.09	0.09	0.60	Demirezen and Uruc [58]
	Romania	–	0.08–0.19 0.16	0.72–0.97 0.82	–	0.71–0.96 0.84	23.1–45.7 38.4	Hoha et al. [1]
	Iran	–	–	0.15–0.70 0.35	–	1.13–3.00 1.88	2.23–6.71 4.61	Sobhanardakani [56]
Würstel	Italy	ND–0.05 0.02	ND–0.06 0.03	0.21–0.68 0.38	0.13–0.32 0.22	1.05–1.31 1.21	4.93–9.23 6.05	This study
Hot dog of beef	Iran	–	0.002–0.01	0.04–0.09	–	–	–	Abedi et al. [59]
Hot dog	Bangladesh	–	0.01	1.01	3.05	110.90	65.55	Chowdhury et al. [60]
Salami	Italy	ND–0.05 0.01	ND–0.06 0.02	0.16–0.49 0.29	0.11–0.29 0.20	0.85–1.35 1.13	5.01–10.18 7.32	This study
	Spain^a^	< 0.100	< 0.025	0.044	–	–	–	Martí-Cid et al. [54]
	Spain	0.013	0.015	0.046	–	–	–	Martorell et al. [53]
	Turkey	–	BDL	0.11	BDL	0.21	12.7	Daşbaşi et al. [57]
	Turkey	–	0.008	0.13	0.09	0.08	0.45	Demirezen and Uruc [58]
	Slovak Republic	0.005–0.009 0.007	–	–	–	–	–	Lukáčová et al. [55]
	Romania	–	0.09–0.24 0.21	0.72–1.06 0.96	–	1.12–1.55 1.32	26.8–55.9 32.19	Hoha et al. [1]
Meat and meat products	Spain	–	–	–	0.87	0.95	43.30	Perelló et al. [61]
	Spain^a^	< 0.100	0.006	0.043	–	–	–	Martí-Cid et al. [54]
	Spain	0.009	0.005	0.065	–	–	–	Martorell et al. [53]
	Sweden	–	0.002	0.004	0.019	0.74	22.50	Becker et al. [62]
	India	–	0.004–0.009	–	0.21–0.27	–	–	Dubey et al. [63]

^a^ng/kg

### Nonessential Metals

There was no significant difference in the concentration of cadmium and mercury (*P* > 0.05), while a significant fluctuation between Pb levels and those of Cd and Hg (*P* < 0.0001) was encountered. Thus, the nonessential metals were detected at concentrations varying in the order Pb > Cd = Hg.

### Lead

The statistical analysis indicated a heterogeneity in concentration levels among different types of processed meats (*P* < 0.002). The highest average concentrations were encountered in würstel (0.38 μg g^−1^), mortadella, and cured sausage (0.34 μg g^−1^), followed by raw ham (0.30 μg g^−1^) and salami (0.29 μg g^−1^), whereas the lowest values were in baked ham (0.22 μg g^−1^). The observed variability is not an unexpected finding, but reflects the large number of factors that influences the metal accumulation in animal products. The contamination is, in fact, a complex matter depending on environment quality in which the animal lives, contamination level in the feed, characteristics of the possible contamination either during the steps of the manufacturing processes or during the packing and storage procedures. More, spices added to processed meats, while promoting flavor and texture to food, may bring further toxic substances [64]. Obviously, all these factors do not allow an easy comparison of the results, but, in general, contamination levels in present study were similar to those encountered by Martorell et al. [53] and Sobhanardakani [56], but higher than those reported by Demirezen and Uruc [58], Abedi et al. [59], Daşbaşi et al. [57] and Marin et al. [52]. According to Commission Regulation (EC) No 2015/1005 [12], the maximum permitted limit for this toxic element in meat is 0.10 μg g^−1^. Evaluating Pb content found in this study, it was observed that it was above the established level in all the samples analyzed, indicating a potential hazard for consumer’s health.

### Cadmium

The fluctuation of the Cd contents was significant among various products (*P* < 0.01). Cd was detected in 75% of the samples examined and contamination results varied from 0.003 to 0.05 μg g^−1^. The highest average concentrations were observed in cured sausage and würstel (0.03 μg g^−1^), while baked and raw ham exhibited the lowest average levels (0.01 μg g^−1^). The low degree of contamination observed in the most of samples examined can be the result of appropriate hygienic practices during pig farming, focused above all on the type and composition of feed. Feeds, in fact, artificially enriched with essential elements to promote optimum growth rate, may also contain toxic elements [65]. In pigs, in particular, a significant increase of cadmium accumulation in tissues has been observed when low calcium content was present in commercial fattening rations [66]. However, our findings were in good agreement with those available in literature for processed meats vended in Bangladesh [60], Spain [52, 53], Iran [59], and Turkey [57]. The European Commission Regulation set a maximum limit of 0.05 μg g^−1^ for this element in muscle meat of terrestrial animals [13]. According to this standard, the concentrations measured were within the permitted limit, though a small sample percentage (6%) showed levels of the same order of magnitude of the authorized limit.

### Mercury

A comparison between Hg concentrations determined in various products did not show differences at the level of significance (*P* > 0.05). Undetectable concentrations were found in 40% of samples examined, while the remaining has levels ranging from 0.01 to 0.05 μg g^−1^. Hg concentrations in this study were in good agreement with data reported in literature [53–55], in which a tendency for a declining mercury content in meat products has already been noted in past decades, probably reflecting either the decrease in environmental burden of Hg or the reduced use of fish products in pig feed [67, 68]. The mean levels in this study were approximately 2 times higher than those obtained in various meat products from Spain [53] and Slovak Republic [55]. The European Commission has not established statutory limits for Hg in meat products, while there are notifications published by Food and Agriculture Organization [48], United States Department of Agriculture [49] and by China government [50] (Table 1). Taking into consideration these safety guidance, all analyzed samples remained within legal margins, although the concentrations in a small sample percentage (9%) resulted equal to the limit of 0.05 μg g^−1^established by China and USDA.

### Essential Metals

There were significant concentration differences among essential metals tested being in the order Zn > Cu > Cr (*P* > 0.0001). As for Hg also for these essential elements, legal thresholds are inexistent in Europe while, as can be read in Table 1, various national and international health organizations have established limits above which the food is considered unsuitable for human consumption.

### Zinc

Significant concentration differences were observed among the various types of processed meats (*P* < 0.04). The average contents varied from a minimum of 5.71 μg g^−1^ in raw ham to a maximum of 7.32 μg g^−1^ in salami, while the other products showed levels of the same order of magnitude (*P* > 0.05). These values were similar to those measured in literature for cured sausage consumed in Iran [56], but lower than those reported in meat products marketed in Turkey [57], Bangladesh [60], Spain [61], and Sweden [62]. For this metal, the European Commission has not established a legal maximum limit in contrast to FAO [51], which have set a limit of 50 μg g^−1^. However, none of the examined samples exceeded this value permitted.

### Copper

The statistical analysis revealed significant differences between the six types of meat products studied (*P* < 0.04). The concentrations varied from a minimum of 0.85 μg g^−1^ encountered in baked ham to a maximum of 1.35 μg g^−1^ in mortadella and salami samples. Compared with literature data, the concentrations in this investigation were approximately of the same order of magnitude to those found in products marketed in Iran [56], Spain [52, 61], and Sweden [62], but higher than those from Turkey [57, 58]. None of the samples in this study had Cu content exceeding the maximum level of 3 μg g^−1^ prescribed by FAO [51].

### Chromium

Significant concentration differences were observed among the various types of processed meats (*P* < 0.004). Chromium was detected in all the samples and varied in the range from 0.07 to 0.32 μg g^−1^. The comparison among the levels revealed the highest average values in cured sausage (0.23 μg g^−1^), followed by würstel (0.22 μg g^−1^), salami, and mortadella having the same contaminant degree (0.20 μg g^−1^) and raw ham (0.19 μg g^−1^), while the lowest levels were in baked ham (0.15 μg g^−1^). Comparing with the results of other studies, the average concentrations obtained here were similar to those reported in processed meat from India [63] and Spain [52], but notably lower than those in hot dogs from Bangladesh [60] and in other meat products from Spain [61]. There are no set standards for concentrations of this element in meat by European Union, while the United States Department of Agriculture *(*USDA) has established a level of 1.0 μg g^−1^ [49], which was not exceeded in any samples in this study.

### Estimated Intake and Risk Assessment

The knowledge of the content of metals in food is of enormous importance for the risk evaluation to human, especially for the metals that are extremely toxic, even in trace amounts. As above reported, the European Union (EU) has set levels for toxic elements with a view to reducing their presence in foods to the lowest levels reasonably achievable so as to secure high levels of public health protection. In this scenario, the Joint FAO/WHO Expert Committee established tolerable intakes to fix the amount of a substance that can be ingested over a lifetime without appreciable health risk. In detail, the Joint FAO/WHO Expert Committee on Food Additives has established Provisional Tolerable Weekly Intakes (PTWIs) of 5 μg kg^−1^ bw week^−1^ and 25 μg kg^−1^ bw week^−1^ for Hg and Pb, respectively [69, 70]. Also for cadmium the Joint FAO/WHO Expert Committee on Food Additives (JEFCA) has set a provisional tolerable monthly intake (PTMI) of 25 μg kg^−1^ body weight. It is preferred to express the intake on a monthly basis because of the long half-life of this metal, daily ingestion in food has little, or even an insignificant effect on the overall exposure [71]. Based on these standards, and considering the mean concentrations, the estimated intakes of trace metals for total population and for children are presented in Table 3. The calculated intakes of Pb and Hg for total population were lower than the tolerable doses, with percentages from 2.5 to 4.2% and from 0.8 to 1.4% of the PTWIs, respectively. With regard to Cd, the monthly intake estimated remained within the established safety margin, with percentages ranging from 0.5 to 1.2% of the PTMI. For children the intakes of Pb, Hg, and Cd were higher than total population but remained below the respective reference values with a percent contribution varying from 5.6 to 9.5%, from 1.4 to 2.8%, and from 1.2 to 2.4%, respectively. Concerning essential metals, their presence in trace amounts within the body is required, because they play an important role in maintenance of numerous physiological processes. For these metals, the daily intakes were compared with the average requirement values of recommended daily allowance (RDA) [39]. Concerning total population, the inspection of the intake data (Table 3) indicated that the consumption of meat products provided 1.4–2.2% of the adequate daily intake of Zn, Cu accounted for 3.3% of RDA, while the estimated intake of Cr constituted from 11.5 to 24.4% of the necessary daily intake. In the case of children, the essential metal intakes through the consumption of these meat products represented 2.6–3.4%, 6.8%, and 23.2–35.1% of the RDA for Zn, Cu, and Cr, respectively (Table 3). These findings revealed that the intakes of Cr from the ingestion of these products were quite high contributing significantly to the dietary requirement of metal. The EDI values obtained have been used to estimate the non-cancer risk through the hazard quotient (HQ), which is considered as the potential risk of adverse health effects from toxic substances to indicate the long-term risk assessment. Concerning total population values of HQ lower than 0.07, 0.01, and 0.004 were obtained for Hg, Cd, and Pb respectively, whereas for essential metals the values ranged from 0.01 to 0.03 μg g^−1^. In children the HQ values for Hg, Cd, and Pb were lower than 0.16, 0.02, and 0.01, respectively, while values between 0.02 and 0.07 were calculated for the essential elements. Also the hazard index (HI), which expresses the cumulative value of the effects of different metal mixtures, was less than 1 for all metals and for both considered groups (total population: 0.10–0.13; children: 0.21–0.30), although the risk index for children was from 2 to 3 times higher than total population (Table 4). Table 5 shows the possibility of exposed subjects developing cancer from lifetime exposure to carcinogenic metals. Due to the lack of carcinogenic slope factors for Hg, Zn, and Cu, only the cancer risks for the other three metals were estimated. In line with US EPA [42], the value of cancer risk in the range of 1 × 10^−6^ to 1 × 10^−4^ is an acceptable or tolerable risk, a risk of less than 1 × 10^−6^ is not regarded to cause significant health effects and thus can be ignored and a risk exceeding 1 × 10^−4^ is perceived as unacceptable. In this context, in the total population, the incremental lifetime risk cancer (ILRC) for Pb was equal to the threshold risk limit in salami (0.98 × 10^−4^); Cd violated the risk in mortadella (1.11 × 10^−4^), würstel (1.48 × 10^−4^), and cured sausage (1.45 × 10^−4^), whereas none of the products tested surpassed the risk limit for Cr (2.88 × 10^−5^–4.36 × 10^−5^). In children ILCR for Cd violated the threshold risk in all the studied meat products (1.39 × 10^−4^–3.42 × 10^−4^), Cr infringed the risk value in cured sausage (3.36 × 10^−4^), würstel (3.42 × 10^−4^), and salami (2.28 × 10^−4^), whereas none of the products crossed the designated risk limit for Pb (1.71 × 10^−6^–2.91 × 10^−6^). The cumulative cancer risk (∑ILCR) findings for total population (0.97 × 10^−4^–1.91 × 10^−4^) indicated that raw and baked ham consumption would result in an excess of 10 cancer cases per 100.000 people, while the highest cancer risk resulting in an excess of 19 cancer cases per 100.000 people derived from würstel and cured sausage consumption. For children, the cumulative cancer risk of all samples studied exceeded the recommended threshold risk (2.25 × 10^−4^–4.42 × 10^−4^) with the consumption of raw and baked ham, cured sausage, and würstel having the lowest (23 cancer cases per 100.000 people) and the highest chances of cancer risk (44 cancer cases per 100.000 people), respectively. Concerning the margin of exposure (MOE) to Pb, the total population intake scenario (8.79–14.2) revealed a very low risk. Similarly in children, all MOE values (1.90–3.07), although lower compared with those calculate in total population, were above the critical limit indicating a negligible health risk (Table 5). However, when assessing exposure, there are a number of scientific uncertainties, including both technical (e.g., sampling procedure, sample preparation, element chemical speciation, variation of consumption values per regions of the same country, food containers) and biological factors (e.g., natural variability in an individual’s response, bioavailability of trace elements after food ingestion, nutritional status) which need to be acknowledged because could lead to bias the results. In our case, it is important to emphasize that in the absence of consumption values relative to each meat product considered, it has been employed a unique ingestion rate (total population: 27.0 g day^−1^, children: 23.3 g day^−1^), parameter that is very influential in determining the outcome of an exposure assessment. In this contest, it is important to point out that HQ of toxic metals could be a cause of concern for children (0.13–0.19) when additional sources of metal exposure, such as consumption of other foodstuffs, dermal contact, and dust inhalation, are taken into account. In a similar way, the MOE values for the Pb exposure from cured sausage consumption in children (1.90) cannot be ignored. This is especially likely to be true in view of the unique vulnerability of children to heavy metal poisoning, especially to those deriving from Pb. Several reasons, including behavioral characteristics such as outdoor activity, less concern for hygiene conditions, hand-to-mouth activities, and a greater predisposition to the absorption of lead from the gastrointestinal tract, make, in fact, children particularly vulnerable to lead poisoning [72].

**Table 3:** Estimated dietary intake for Pb, Cd, Hg (μg kg^−1^ body weight) and for Zn, Cu (mg day^−1^) and Cr (μg day^−1^) through pork meat products consumption for total population and children plus food standards (% PTWI, PTMI and RDA)

	Total population											
Meat products	Pb		Cd		Hg		Zn		Cu		Cr	
	EDI^a^	% PTWI^b^	EDI	% PTMI^c^	EDI	% PTWI	EDI	% RDA^d^	EDI	% RDA	EDI	% RDA
Baked ham	0.09	2.5	0.005	0.6	0.005	0.8	0.16	1.5 (m); 1.8 (w)	0.03	3.3	4.03	11.5 (m); 16.1 (w)
Raw ham	0.11	3.1	0.004	0.5	0.005	0.8	0.15	1.4 (m); 1.7 (w)	0.03	3.3	5.05	14.4 (m); 20.2 (w)
Mortadella	0.13	3.6	0.01	1.2	0.01	1.4	0.16	1.5 (m); 1.8 (w)	0.03	3.3	5.29	15.1 (m); 21.2 (w)
Cured sausage	0.13	3.6	0.01	1.2	0.01	1.4	0.16	1.5 (m); 1.8 (w)	0.03	3.3	5.88	16.8 (m); 23.5 (w)
Würstel	0.15	4.2	0.01	1.2	0.01	1.4	0.17	1.5 (m); 1.9 (w)	0.03	3.3	6.11	17.5 (m); 24.4 (w)
Salami	0.11	3.1	0.01	1.2	0.01	1.4	0.20	1.8 (m); 2.2 (w)	0.03	3.3	5.43	15.5 (m); 21.7 (w)
	Children											
Baked ham	0.20	5.6	0.01	1.2	0.01	1.4	0.14	2.8	0.03	6.8	3.48	23.2
Raw ham	0.27	7.6	0.01	1.2	0.01	1.4	0.13	2.6	0.03	6.8	4.35	29.0
Mortadella	0.30	8.4	0.02	2.4	0.02	2.8	0.14	2.8	0.03	6.8	4.57	30.5
Cured sausage	0.31	8.7	0.02	2.4	0.01	1.4	0.14	2.8	0.03	6.8	5.27	35.1
Würstel	0.34	9.5	0.02	2.4	0.02	2.8	0.14	2.8	0.03	6.8	5.07	33.8
Salami	0.26	7.3	0.02	2.4	0.01	1.4	0.17	3.4	0.03	6.8	4.68	31.2

*m* men; *w* women

^a^*EDI* Estimated daily intake; ^b^*PTWI* provisional tolerable weekly intake (Pb: 25 μg g^−1^ body weight; THg: 5.0 μg g^−1^ body weight) [36]; ^c^*PTMI* provisional tolerable monthly intake (Cd: 25 μg g^−1^ body weight) [71]; ^d^*RDA* recommended daily allowance [39] (Zn: men 11 and women 9 mg day^−1^; Cu: 0.9 mg day^−1^; Cr: men 35 and women 25 μg day^−1^)

**Table 4:** Hazard quotient (HQ) and hazard index (HI) of the studied metals through consumption of pork meat products for total population and children

Meat products	HQ (total population)						HI
	Pb	Cd	Hg	Zn	Cu	Cr	
Baked ham	0.002	0.005	0.05	0.01	0.01	0.02	0.10
Raw ham	0.003	0.004	0.05	0.01	0.01	0.02	0.10
Mortadella	0.004	0.01	0.07	0.01	0.01	0.03	0.13
Cured sausage	0.004	0.01	0.05	0.01	0.01	0.03	0.11
Würstel	0.004	0.01	0.07	0.01	0.01	0.03	0.13
Salami	0.003	0.01	0.05	0.01	0.01	0.03	0.11
	HQ (children)						HI
Baked ham	0.01	0.01	0.11	0.02	0.02	0.04	0.21
Raw ham	0.01	0.01	0.11	0.02	0.02	0.06	0.24
Mortadella	0.01	0.02	0.16	0.02	0.03	0.06	0.30
Cured sausage	0.01	0.02	0.12	0.02	0.02	0.07	0.26
Würstel	0.01	0.02	0.15	0.02	0.03	0.06	0.29
Salami	0.01	0.02	0.12	0.02	0.03	0.06	0.26

**Table 5:** Incremental lifetime cancer risks (ILCRs) through consumption of pork meat products for total population and children

	Total population				
Meat products	ILCR Pb	MOE Pb	ILCR Cd	ILCR Cr	∑ILCR
Baked ham	7.37 × 10^−7^	14.20	7.37 × 10^−5^	2.88 × 10^−5^	1.03 × 10^−4^
Raw ham	9.73 × 10^−7^	12.01	6.00 × 10^−5^	3.60 × 10^−5^	0.97 × 10^−4^
Mortadella	1.12 × 10^−6^	10.34	1.11 × 10^−4^	3.78 × 10^−5^	1.50 × 10^−4^
Cured sausage	1.26 × 10^−6^	8.79	1.48 × 10^−4^	4.20 × 10^−5^	1.91 × 10^−4^
Würstel	1.12 × 10^−6^	10.70	1.45 × 10^−4^	4.36 × 10^−5^	1.90 × 10^−4^
Salami	0.98 × 10^−4^	12.20	0.97 × 10^−6^	3.88 × 10^−5^	1.38 × 10^−4^
	Children				
Baked ham	1.71 × 10^−6^	3.07	1.71 × 10^−4^	6.67 × 10^−5^	2.39 × 10^−4^
Raw ham	2.25 × 10^−6^	2.59	1.39 × 10^−4^	8.34 × 10^−5^	2.25 × 10^−4^
Mortadella	2.59 × 10^−6^	2.23	2.56 × 10^−4^	8.75 × 10^−5^	3.46 × 10^−4^
Cured sausage	2.59 × 10^−6^	1.90	3.36 × 10^−4^	1.01 × 10^−4^	4.40 × 10^−4^
Würstel	2.91 × 10^−6^	2.31	3.42 × 10^−4^	0.97 × 10^−4^	4.42 × 10^−4^
Salami	2.23 × 10^−6^	2.64	2.28 × 10^−4^	0.90 × 10^−4^	3.20 × 10^−4^

## Conclusions

The obtained set of data represents a first survey of essential and toxic elements in a pork meat product variety. Differences either in the levels of metal accumulation or in function of different products tested have been encountered. The content of Pb in all samples is higher than the limit established by European Union legislation, whereas in a small number of samples Cd and Hg concentration is higher than the allowed limits. However, the estimated intake values showed that the intakes of metals from the ingestion of these products were within their provisional tolerable daily intake limits for the toxic metals and recommended daily intake values for the essential metals in both tested groups. Concerning the risk assessment, the HQ values of the individual metals indicated that there is no health risk, but their combined effects might constitute a potential risk for children. Furthermore, the cumulative cancer risk (ƩILCR) of all samples studied exceeded the recommended threshold risk limit (> 10^−4^) indicating a risk of potential health problems for consumers, especially for children, sensitive group of population. Concluding the present study indicates the need to monitor contamination with heavy metals in these products on regular basis and at larger scale.
